# Acute non-hemolytic transfusion reactions and HLA class I antibody: advantages of solid phase assay compared with conventional complement-dependent assay

**DOI:** 10.1111/j.1365-3148.2009.00972.x

**Published:** 2010-04

**Authors:** S Imoto, K Kawamura, Y Tokumine, N Araki, S Akita, C Nishimura, H Inaba, K Saigo, O Mabuchi, H Okazaki

**Affiliations:** 1 Japanese Red Cross Hyogo Blood CenterKobe, Japan; 2 Japanese Red Cross Society, Blood Service Headquarters, Central Blood InstituteTokyo, Japan; 3 Itami General HospitalItami, Japan; 4 Japanese Red Cross Himeji HospitalHimeji, Japan; 5 Japanese Red Cross Osaka Blood CenterOsaka, Japan; 6 Himeji Dokkyo UniversityHimeji, Japan

**Keywords:** acute non-hemolytic transfusion reactions, febrile reaction, HLA Class I antibodies, recombinant HLA molecules, solid phase assays, TRALI

## Abstract

To evaluate the specific reactivity of HLA Class I antibodies (HLA-I Abs) in acute non-hemolytic transfusion reactions (ANHTRs) using solid phase assays (SPAs) and conventional complement-dependent lymphocyte cytotoxicity test (LCT). ANHTRs are major issues in transfusion medicine. Anti-leukocyte antibodies have been implicated as one of the causative agents of transfusion-related acute lung injury (TRALI) and febrile reaction. Antibodies to HLA Class I and/or Class II (HLA Abs) have been intensively studied using SPAs for TRALI, but not for febrile reaction. About 107 patients and 186 donors associated with ANHTRs were screened for HLA Abs by SPAs such as enzyme-linked immunosorbent assay (ELISA) and the Luminex method. When HLA-I Ab was detected, its specific reactivity was evaluated by comparing its specificity identified by the Luminex method using recombinant HLA molecules and cognate HLA antigens (Ags), as well as LCT with or without anti-human globulin (AHG). The incidences of HLA Abs were as high as 32·7% of patients' serum samples and 16% of donors' serum samples. The incidence of HLA-I Abs did not differ significantly between cases of febrile and allergic reactions. However, HLA-I Abs associated with febrile reaction showed a significantly higher rate of possessing specific reactivity to cognate HLA Ags than those associated with allergic reactions. In addition, the Luminex method enabled the detection of HLA-I Abs much earlier than AHG-LCT in serum samples from a patient with febrile reaction and platelet transfusion refractoriness (PTR). SPAs seem more useful than AHG-LCT for evaluating reactivity of antibodies in ANHTR cases.

After a marked decrease in the incidence of transfusion-transmitted infectious events, acute non-hemolytic transfusion reactions (ANHTRs) have become the major issues (Combs *et al.*, 2002; Klein & Anstee, 2005). ANHTRs range from mild urticaria, febrile reaction to the most life-threatening transfusion-related acute lung injury (TRALI) (Silliman *et al.*, 2003; Bux 2005). Anti-leukocyte antibodies have been implicated as one of the causative agents of TRALI and febrile reaction (Thompson *et al.*, 1971; Popovsky & Moore, 1985; Payne R, 1957; de Rie *et al.*, 1985). Among anti-leukocyte antibodies, antibodies to human leukocyte antigen (HLA) Class I and/or Class II (HLA Abs) are much more frequently detected than anti-granulocyte antibodies (Bux *et al.*, 1992; Kopko *et al.*, 2003; Zupanska *et al.*, 2007). Sensitive solid phase assays (SPAs) using plastic plates [enzyme-linked immunosorbent assay (ELISA)] or microbeads (flow cytometry or Luminex method) are commercially available for HLA Abs (Stroncek *et al.*, 2007). For TRALI, HLA Abs have been intensively analyzed using SPAs, but for febrile reaction, SPAs have not been used except in our previous study (Payne R, 1957; Decary *et al.*, 1984; de Rie *et al.*, 1985; Imoto *et al.*, 2007). TRALI is frequently accompanied by fever. As Davis *et al*. have recently reported in ‘a Touch of TRALI’, some of the TRALI cases may have been classified into febrile reaction when the pulmonary manifestation was less severe (Davis *et al.*, 2008). SPA-based analysis of HLA Abs not only for TRALI, but also for febrile reaction and allergic reactions may provide useful information for understanding the role of HLA Abs.

Platelet transfusion refractoriness (PTR) is also an important issue in transfusion medicine. HLA-I Abs in patients' sera have been implicated in most of the PTR cases. For pateitns with PTR, screening of donor(s) for HLA-compatible platelet concentrates (HLA-PCs) is necessary as soon as possible.

We in a regional blood service center of the Japanese Red Cross Society (JRCS) have studied ANHTRs (Imoto *et al.*, 2007). Moreover, we have promoted the registration of donors for HLA-PCs. SPA-based analyses gave us high HLA Abs positivity rates, 36% in female patients' sera and 13% in female donors' sera associated with ANHTRs, whereas no significant difference was observed between allergic and febrile reactions (Imoto *et al.*, 2007). The results suggest that most of the HLA Abs were coincidentally detected but not causative. To evaluate the role of detected HLA Abs, their specific reactivity to cognate HLA Ags should be examined. In this study, by utilizing the HLA-typing data of donors for HLA-PCs, we were able to evaluate the specific reactivity of HLA-I Abs detected in 16 serum samples associated with 12 cases of ANHTRs. The specificity of the HLA-I Abs was identified by the Luminex method using recombinant HLA molecules, LABScreen® Single Antigen. The specific reactivity of the HLA-I Abs to the cognate HLA Ags was evaluated by comparing the specificity of the antibodies and the cognate HLA -A and -B loci, as well as by conventional AHG-LCT. We examined only the specific reactivity of HLA-I Abs, because the HLA-typing data of donors were for HLA-PCs; thus, data only for HLA Class I loci were available. We also present a PTR case with several episodes of febrile as well as allergic reactions, for which we were able to sequentially analyze HLA-I Abs.

## MATERIALS AND METHODS

### Patients, blood products and donors

From 12 March 2004 to 26 December 2005, 107 cases of ANHTRs (56 males and 51 females) were voluntarily reported from 36 hospitals located in Hyogo prefecture. ANHTRs in all of these cases occurred within 24 h of transfusion. There were 65 cases of allergic reactions (ranging from urticaria to anaphylactic), 28 cases of febrile reaction and 14 cases of TRALI/dyspnoea (dyspnoea or hypoxia not due to bronchospasm. See Imoto *et al.*, 2007 for details). About 186 blood components were involved; 77 red blood cell concentrates (RCCs: 400-mL-whole-blood-derived, 66; 200-mL-whole-blood-derived, 11), 57 platelet concentrates (PCs; all were single-donor-apheresis-derived) and 52 fresh frozen plasma (FFP: 400-mL-whole-blood-derived, 38; single-donor-apheresis-derived, 14). The 186 blood components were derived from 126 male (61·2%) and 60 female (38·8%) donors.

For all the 107 patients, sera were obtained from the physicians after obtaining their informed consent. For 131 of the 186 donors of blood products associated with ANHTRs, serum samples were prepared from blood products in segment tubes obtained from physicians. Before antibody detection, a donor's plasma sample was defibrinated by treating with thrombin and calcium chloride (Araki *et al.*, 1995).

### Assay of HLA Abs

ELISA (Lambda Antigen Tray™ (LAT); One Lambda, Inc., Canoga Park, CA, USA) was used from March 2004 to September 2004 and the Luminex method from October 2004 to December 2005, in accordance with the manufacturer's instructions. For ELISA, LAT-M was used for screening and when the reaction was positive, the specificity of the antibody was identified using another panel antigen tray, LAT-1240. For donor–patient pair analysis, the specificity was determined by the Luminex method using LABScreen® Single Antigen (One Lambda). For the Luminex method, after incubation with color-coded beads (LABScreen PRA Class I and Class II), fluorescent emission from the beads was detected and analyzed using a Luminex 100 IS System (Luminex Corporation, Austin, TX, USA). The antibody specificity to HLA -A, and -B alleles was determined using LABScreen® Single Antigen.

### HLA allele typing

When HLA-I Ab was detected in a donor's serum, HLA typing of the patient was performed. Blood collected into an EDTA-2Na-containing tube was obtained from the physician after obtaining the patient's informed consent. DNA typing of the HLA -A, -B and -C loci was performed by the PCR-SSOP-Luminex method using Genosearch HLA -A, -B and -C (MBL, Nagoya, Japan) with a Luminex 100™ IS fluoroanalyzer (Itoh *et al.*, 2005), in accordance with the manufacturer's instructions. Data on HLA-typing by the same method were available for donors who had been registered for HLA-PC donation.

### Crossmatching with AHG-LCT

Peripheral blood lymphocytes (PBLs) were separated by centrifugation of EDTA-2Na-anticoagulated blood over a Ficoll-Conray gradient. For donors who had been registered for HLA-PCs, lymphocytes were frozen-stored, and were prepared by thawing before use. Crossmatching between HLA-I Ab-positive sera and cognate lymphocytes was carried out by LCT and AHG-LCT as previously described (Araki *et al.*, 1995). Briefly, lymphocytes were mixed with serum and incubated for 30 min at 22°C. Rabbit complement (Class I Complement; One Lambda) was added and the mixture was incubated for 60 min at 22°C. After addition of 2% eosin, the percentage of damaged cells was evaluated under a microscope. The decision of positivity was made when more than 20% of the cells were stained with eosin.

## RESULTS

### Incidence of HLA Abs in patients and donors

About 107 cases of ANHTRs consisting of 65 cases of allergic reactions, 28 cases of febrile reaction and 14 cases of TRALI/dyspnoea were analyzed. HLA Abs were detected in 35 (32·7%) patients, 10 of the 56 (17·9%) males and 25 of the 51 (49%) females. Of the 10 antibody-positive male patients, 5 possessed antibodies to HLA Classes I and II, four to Class I and one to Class II. Of the 25 antibody-positive female patients, 19 possessed antibodies to HLA Classes I and II, 5 to Class I, and 1 to Class II. Taken together, 33 patients possessed HLA-I Abs. The positivity rates of HLA-I Ab were 30·8% for allergic reactions, 39·2% for febrile reactions and 21·4% for TRALI/dyspnoea. The differences were statistically not significant.

The 131 (93 males and 38 females) donors were examined. HLA Abs were detected in 16% of the donors (10·8% of males and 28·9% of females). Of the 10 antibody-positive male donors, three possessed antibodies to HLA Class I and II, 6 to Class I, and 1 to Class II. Of the 11 antibody-positive female donors, 3 possessed antibodies to HLA Classes I and II, 5 to Class I, and 3 to Class II. Taken together, 17 donors possessed HLA-I Abs.

In total, 47 ANHTR cases were associated with HLA-I Abs either in patients' or donors' sera.

### Evaluation of specific reactivity of HLA-I Abs

Among the 33 cases with HLA-I-Ab-positive patients, HLA-typing data of the donors were available for seven cases. Among the 13 ANHTR cases with 17 HLA-I-Ab-positive donors, we were able to examine HLA-typing data of the patients for 9 cases. As a result, the specific reactivity of the HLA-I Abs to cognate HLA Ags was examined for 16 donor–patient pairs of the 12 ANHTR cases, consisting of 7 allergic reactions, 4 febrile reaction, and 1 suspected case of TRALI (Tables 1 and 2). Cases 5 and 6 occurred in the same female patient at different times. Case 10 was a suspected case of TRALI. The patient was a 6-year-old boy with congenital deformity of the right lung lobe and transposition of great artery. During cardiovascular surgery, he developed severe hypoxia 90 min after starting transfusion. Cardiac failure was excluded. Chest radiography showed pulmonary edema of the left lung and atelectasis of the right lung owing to the congenital deformity. Although the pulmonary oedema was unilateral, TRALI was suspected clinically.

**Table 2:** Reactivity of the HLA Class I antibodies

Case	Type of ANHTR	HLA antibodies in patient's serum	HLA antibodies in donor's serum	Serum tested (gender)	Specificity of the HLA Class I antibodies	HLA Class I type of the corresponding lymphocytes	Reactive antigen	Crossmatches by AHG-LCT
1	Allergic	Negative	Class I	Donor's serum (female)	B7, B73, B55 (^*^5501), B27, B42, B60, B81, B48, B61, B41	Patient: A2/11·1, B35/55·1(^*^5502), Cw1/9	Nothing	Negative
2	Allergic	Negative	Class I	donor's serum (male)	A68, A2, B45, B77, B76	Patient: A26/-, B46/61, Cw1/10	Nothing	Negative
3	Febrile	Class I & II	Negative	Patient's serum (female)	A11, B60(^*^4001), B48, B45, B50, B81, B41, B49	Donor: A^*^2402/-, B^*^0702/^*^4001(B60), Cw^*^0401/^*^0702	B60(^*^4001)	Negative
4	Allergic	Class I & II	Negative	Patient's serum (female)	A11, A24, A3, A32, A23, A25, A30, A31, A68, A2, A69, A74, B49, B52, B27, B51, B53, B38, B57, B77, B47, B37, B58, B44, B13, B76	Donor: A26/-B61/-, Cw10/-	Nothing	Negative
5	Febrile	Class I & II	ND	Patient's serum (female)	B39, **B54**, B55, B7 B27, B42, B49, B41, B62, B56, B57, B67, B8201, B73, B38, B81, B18, B37, B45, B50, B58, B64, A68, B8, A30, A31, B72	Donor: A24/31, B35/**54**, Cw1/9	**B54**	**ND**
6	Allergic	Class I & II	Negative	Patient's serum (female)	B54, B55, B7, B42, B39, B27, B41, B56, B49, B67, B57, B62, B37, B8201, B81, B58, B45, B38, B50, B73	Donor: A2/-, B51/60, Cw14/15	Nothing	Negative
7	Febrile	Negative	Class I	Donor's serum (female)	**A24**, A32, A25, A23, B49, B52, B51, B53, B27, B73, B38, B77, B78, B57, B37, B46, B58, B35, B18	Patient: A**24**/31, B54/60, Cw1/10	**A24**	**positive**
8	Allergic	Class I & II	Class I	Patient's serum (male)	A11, A69, A68, A3, A2, A30, **A31**, A74, A32, A24, B57, B55, B7, B58, B45, B42, B54, B27, B81, B44, B67	Donor: A24/**31,** B48/54, Cw4/8	**A31**	Negative
			Class I	Donor's serum (male)	A2, A68, A69, B13, B67	Patient: A24/26, B52/60, Cw4/10	Nothing	Negative
9	Allergic	Class I & II	Class I	Patient's serum (female)	A1, A36, B7	Donor: A31/-, B52/61, Cw3/-	Nothing	Negative
			Class I	Donor's serum (female)	**A24**, B7, B42, B27, B55, B60, B81	Patient: A11/^*^2402, B46/51, Cw1/-	**A24**	Negative
10	Possible TRALI	Negative	Class I	Donor's serum (male)	A1	Patient: A2/11, B48/70, Cw7/-	Nothing	Negative
			Class I	donor's serum (male)	B38, B39	Patient: A2/11, B48/70, Cw7/-	Nothing	Negative
			Class I	Donor's serum (female)	A80, B45, B4005, B65, **B48,** B38, B60, B44	Patient: A2/11, B**48**/70, Cw7/-	**B48**	Negative
11	Febrile	Class I & II	Negative	Patient's serum (female)	A30, A3, **A31**, A32, A25, A68, A11, A74, A43, A66, A69, A33, A26, B51, B54, B73	Donor: A24/**31,** B7/60, Cw7/10	**A31**	**positive**
12	Allergic	Class I	Class I	Donor's serum (male)	B57, B58	Patient: A24/33, B44/52, Cw12/14	Nothing	Negative

**Table 1:** Characteristics of patients with ANHTRs

Case	Patient's age	Patient's gender	Disease	Type of transfusion reaction	Blood component	Donor's gender	Leukoreduction*
1	68	Male	Acute myeloid leukemia	Allergic (skin eruption)	RCC	Female	Yes bedside
2	48	Female	NK cell lymphoma	Allergic (skin eruption)	PC	Male	Yes bedside
3	66	Female	Acute myeloid leukemia	Febrile (fever, chills)	HLA-PC	Male	Yes bedside
4	31	Female	Aplastic anemia	Allergic (urticaria)	HLA-PC	Male	Yes prestorage
5	27	Female	Acute myeloid leukemia	Febrile (fever)	PC	Male	Yes prestorage
6	27*	Female	Acute myeloid leukemia	Allergic (urticaria)	HLA-PC	Male	Yes prestorage
7	67	Male	Non-Hodgkin's lymphoma	Febrile (fever, chills)	PC	Female	Yes prestorage
8	78	Male	Acute myeloid leukemia	Allergic (urticaria)	PC	Male	Yes prestorage
9	58	Female	Gastric cancer	Allergic (urticaria)	RCC	Female	no
10	6	Male	Congenital cardiovascular anomaly (transposition of great artery, deformity of the right lung)	TRALI s/o (severe hypoxia, pulmonary oedema, mechanical ventilation required)	RCC 5 bags		Yes bedside
					FFP 6 bags	Male 7	no
					PC 1 bag	Female 5	Yes prestorage
11	68	Female	Chronic myelomonocytic leukemia	Febrile (fever, chills)	PC	Male	Yes prestorage
12	50	Male	Gastric cancer	Allergic (urticaria)	PC	Male	Yes prestorage

* Leukoreduction: Bedside indicates usage of leukoreduction filter at bedside. Prestorage indicates leukoreduction at blood center.

Specificity of HLA-I Abs identified using LABScreen Single Antigen was compared with the cognate HLA -A and -B loci (Table 2). For example, in Case 3, the antibody in the patient's serum showed specificity to A11, B60 (*4001), B48, B45, B50, B81, B41 and B49. The donor's HLA type was A*2402/-, B*0702/*4001 (B60), Cw4/7. As the antibody showed specificity to one of the donor's alleles, B60 (*4001), the antibody was determined as having specific reactivity. Specific reactivity was detected in seven cases, consisting of two cases of allergic reactions, four cases of febrile reaction, and one suspected case of TRALI. For the suspected case of TRALI, 12 blood products were transfused. HLA-I Abs were detected in three of the 12 blood products. One of them showed specificity to the patient's HLA allele, B48, and was considered as having specific reactivity. Results showed that 100% of the cases of febrile reaction and the suspected case of TRALI were associated with HLA -I Abs having specific reactivity, whereas only 28·6% (2/7) of the cases of allergic reactions were. The difference was statistically significant (Fisher's exact test, *P* = 0·0278).

The specific reactivity of HLA-I Abs was also tested by lymphocyte crossmatching by AHG-LCT in 15 of the 16 donor–patient pairs (Table 2). Case 5 was not tested because of the poor viability of the donor's lymphocytes. Sera from only two of the 15 pairs showed positive reactions, both of which showed febrile reaction.

### Sequential measurement of HLA-I Ab in a patient with ANHTRs and PTR

**Fig. 1:**
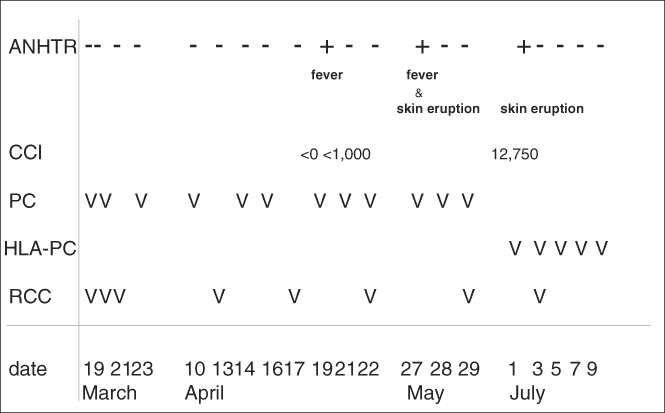
Clinical course of patient with ANHTRs and PTR. Timing of transfusions of PC, HLA-PC and RCC is shown. ‘V’ in the figure indicates transfusion of one blood component, ‘−’ indicates transfusion without ANHTRs and “+” indicates occurrence of ANHTRs. The type of ANHTR is shown under the mark.

Cases 5 and 6 occurred in the same patient at different times (Tables 1 and 2). The patient was a 27-year-old female with acute myeloid leukemia. She had two histories of pregnancy. During her clinical course, she developed fever and skin eruption after PC transfusions, and also developed PTR (Fig. 1).

**Fig. 2:**
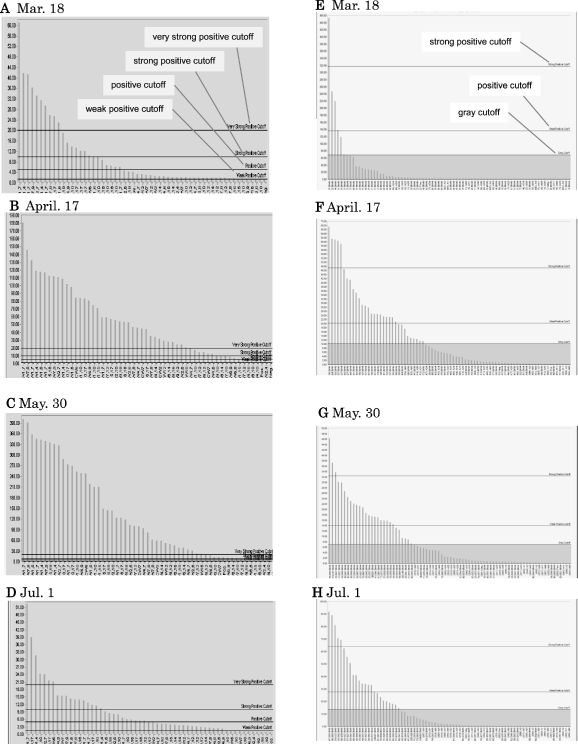
HLA-I Abs measured using LABScreen PRA and Single Antigen. The serum samples from the patient with acute myeloid leukemia collected at four time points were analyzed using LABScreen PRA (A, B, C, D) to examine the range and intensity of reactivity and using LABScreen Single Antigen (E, F, G, H) to determine antibody specificity. Microbeads coated with HLA Class I molecules were laterally allocated in order of reaction intensity. In graphs A–D, the uppermost line indicates ‘very strong positive cutoff’, the second line ‘strong positive cutoff’, the third line ‘positive cutoff’, the fourth line ‘weak positive cutoff’, as indicated in Fig. 2A. In graphs E–H, the upper line indicates ‘strong positive cutoff’, the middle line ‘weak positive cutoff’, the lower line ‘gray cutoff’, as indicated in Fig. 2E.

HLA-I Ab was sequentially measured using the Luminex method (Fig. 2). HLA-I Ab was already detected using LABScreen PRA in a serum sample collected on March 17, before the first transfusion.

Antibody specificity was measured using LABScreen Single Antigen (Fig. 2E–H). The first febrile reaction occurred on April 19. Corrected count increment (CCI) was less than 1000, indicating PTR (Fig. 1, Case 5 in Table 1). The donor's HLA type was A24/31, B35/54, Cw14/15 and serum collected on April 17 showed specificity to B54, one of the donor's HLA alleles, showing specific reactivity (Case 5 in Table 2). The second febrile reaction occurred on May 27, accompanied by skin eruption. The antibody showed increased reaction intensity and reaction range, and the serum sample collected on May 30 showed the highest reaction intensity with the widest reaction range (Fig. 2A–C).

After May 30, HLA-PC was transfused every time, and thereafter, she did not suffer from febrile reaction. The antibody in the serum samples collected on July 1 showed a lower reaction intensity and narrower reaction range (Fig. 2D). However, she developed skin eruption on July 1 after HLA-PC transfusion (Case 6 in Tables 1 and 2, Fig. 1). Platelet recovery was normal (CCI at 1 h was 12,750). The antibody showed no specificity to the corresponding donor's HLA alleles (Table 2 and Fig. 2H), suggesting that the antibody was not the causative agent of the allergic reaction.

When the patient's sera were tested by AHG-LCT using panel lymphocytes, serum samples collected on May 30 showed positive reactions, but serum samples collected on March 17 showed negative reactions. Serum samples collected on April 17 showed non-specific reactions.

## DISCUSSION

Using ELISA and the Luminex method, we observed high incidences of HLA antibodies in both patients and donors associated with ANHTRs. HLA Abs were detected in 32·7% (49% of females and 17·9% of males) of the patients' serum samples and in 16% (28·9% of females and 10·8% of males) of the donors' serum samples. HLA-I Abs were positive in 30·8% of the patients' sera. Other investigators have reported similar results (Fadeyi *et al.*, 2008; Powers *et al.*, 2008). Powers and co-workers screened sera from donors to the hospital blood bank by LABScreen mixed assay, and detected HLA Abs among 42·5% (211/497) of female donors with a history of pregnancy and 12·0% (3/25) of male donors with a history of transfusion (Powers *et al.*, 2008). Densmore and co-workers reported a lower percentage probably because of a lower sensitivity of their assay method, AHG-LCT (Densmore *et al*. 1999).

In our present study, the positivity rates of HLA-I Ab in febrile reaction and allergic reactions were 39·2 and 30·8%, respectively. The difference was statistically not significant. However, the rate of positive specific reactivity of the HLA-I Abs which was determined by comparing the specificity of HLA-I Ab and the cognate HLA -A and -B loci, was significantly higher in febrile reaction (100%) than in allergic reactions (28·6%). In contrast, crossmatching by AHG-LCT showed positive reactions only in 13·3% of ANHTRs. Our results show that HLA-I Abs detected in febrile reaction are more often causative than those detected in allergic reactions, and that SPAs were more sensitive than AHG-LCT. Evaluation of the specific reactivity of HLA Abs by comparing the antibody's specificity and the cognate HLA loci instead of LCT has been increasingly utilized in the field of organ transplantations (Pei *et al.*, 2003; Vaidya *et al.*, 2006; Gupta *et al.*, 2008), as well as in unrelated cord blood transplantations (Takanashi *et al.*, 2008).

Regarding TRALI, we were able to evaluate only one suspected case. HLA-I Abs were detected in 3 of the 12 blood components, one of which showed specificity to the patient's HLA Ag, whereas crossmatching by AHG-LCT showed negative results. Identification of the donor implicated in TRALI seems important to prevent further occurrence of TRALI (Kleinman *et al.*, 2004; Eder *et al.*, 2007; Stroneck & Klein 2007). Our result shows that SPA-based evaluation of specific reactivity of HLA-I Abs is better than AHG-LCT to identify the implicated donors in suspected cases of TRALI.

For a PTR case, we were able to sequentially analyze HLA-I Ab. HLA-I Ab was detected by LABScreen PRA much earlier than by AHG-LCT. This case demonstrates the relationship between the specific reactivity of HLA-I Ab and the occurrence of febrile reaction. In addition, data on antibody specificity seemed useful for selection of HLA-PC donors. Sato and co-workers reported the cases of two PTR patients whose anti-HLA-I Abs were detected by FlowPRA but not by AHG-LCT (Sato *et al.*, 2005). The patients were able to obtain HLA-PCs much earlier than expected by AHG-LCT. Although AHG-LCT has been used as a standard assay method to select donors for HLA-PC, SPA-based methods seem to be more beneficial.

In summary, using SPA-based methods, we were able to observe a significantly higher rate of positive specific reactivity of HLA-I Abs in febrile reaction than in allergic reactions. SPA-based methods seem to be more beneficial than AHG-LCT to identify implicated donors in suspected cases of TRALI, and for selecting compatible HLA-PC donors for patients with PTR. However, the number of evaluated cases was small, and we were not able to evaluate the specific reactivity of co-existing HLA Class II Abs, whereas we confirmed the negativity for anti-granulocyte antibodies examined by previously described methods (Imoto *et al.*, 2007). Further investigation is necessary to confirm our conclusion.
